# Molecular Differences between Squamous Cell Carcinoma and Adenocarcinoma Cervical Cancer Subtypes: Potential Prognostic Biomarkers

**DOI:** 10.3390/curroncol29070372

**Published:** 2022-07-05

**Authors:** Alma D. Campos-Parra, Milagros Pérez-Quintanilla, Antonio Daniel Martínez-Gutierrez, Delia Pérez-Montiel, Jaime Coronel-Martínez, Oliver Millan-Catalan, David Cantú De León, Carlos Pérez-Plasencia

**Affiliations:** 1 Laboratorio de Genómica, Instituto Nacional de Cancerología (INCan), Mexico City 14080, Mexico; adcamposparra@gmail.com (A.D.C.-P.); maga94@comunidad.unam.mx (A.D.M.-G.); oliver.millan.sg@gmail.com (O.M.-C.); 2 Unidad de Investigaciones Biomédicas en Cancer, Instituto Nacional de Cancerología (INCan), Universidad Nacional Autónoma de México (UNAM), Av San Fernando 22, Col. Sección XVI, Mexico City 14080, Mexico; dra.milagros@onco-gine.com (M.P.-Q.); quiechc8@hotmail.com (J.C.-M.); 3 Departamento de Patología, Instituto Nacional de Cancerología (INCan), Mexico City 14080, Mexico; madeliapmg@hotmail.com; 4 Unidad de Biomedicina, Facultad de Estudios Superiores Iztacala, Universidad Nacional Autónoma de México (UNAM), Tlalnepantla de Baz 54090, Mexico

**Keywords:** cervical cancer, adenocarcinoma, squamous cell carcinoma

## Abstract

The most frequently diagnosed histological types of cervical cancer (CC) are squamous cell carcinoma (SCC) and adenocarcinoma (ADC). Clinically, the prognosis of both types is controversial. A molecular profile that distinguishes each histological subtype and predicts the prognosis would be of great benefit to CC patients. Methods: The transcriptome of CC patients from The Cancer Genome Atlas (TCGA) was analyzed using the DESeq2 package to obtain the differentially expressed genes (DEGs) between ADC and SCC. The DEGs were validated on a publicly available Mexican-Mestizo patient transcriptome dataset (GSE56303). The global biological pathways involving the DEGs were obtained using the Webgestalt platform. The associations of the DEGs with Overall Survival (OS) were assessed. Finally, three DEGs were validated by RT-qPCR in an independent cohort of Mexican patients. Results. The molecular profiles of ADC and SCC of the CC patients of the TCGA database and the Mexican-Mestizo cohort (GSE56303) were determined obtaining 1768 and 88 DEGs, respectively. Strikingly, 70 genes were concordant—with similar Log2FoldChange values—in both cohorts. The 70 DEGs were involved in IL-17, JAK/STAT, and Ras signaling. Kaplan-Meier OS analysis from the Mexican-Mestizo cohort showed that higher GABRB2 and TSPAN8 and lower TMEM40 expression were associated with better OS. Similar results were found in an independent Mexican cohort. Conclusions: Molecular differences were detected between the ADC and SCC subtypes; however, further studies are required to define the appropriate prognostic biomarker for each histological type.

## 1. Introduction

Despite early screening programs, cervical cancer (CC) is an unresolved health issue. Although its incidence has decreased by approximately 40%, it is currently the fourth leading cause of cancer-related death in women, accounting for an estimated 341,000 deaths worldwide [1]. Unfortunately, in developing countries, most tumors are diagnosed at advanced clinical stages (75–90%), at which point the tumors are often unresponsive to standard treatment; thus, many CC patients die due to disease recurrence or progression [2]. The most frequent histological types of CC are squamous cell carcinoma (SCC), which accounts for approximately 75–90% of CCs, followed by adenocarcinoma (ADC), which accounts for approximately 10–25% of all CC cases [3]. It is well-described that persistent infection with high-risk human papillomavirus (HR-HPV) types 16, 18, 45, 31, 33, 52, 58, and 35 is the causal factor in the development of CC [4]. Strikingly, several reports have described that HPV16 is more frequent in SCCs, and HPV18 and HPV45 in ADCs [5,6,7]. These histologic types are relevant in terms of patient prognosis; according to National Comprehensive Cancer Network (NCCN) guidelines, both are usually treated similarly, i.e., treatment is based on surgery for early disease and chemoradiotherapy for advanced disease, with survival outcomes for both histologic types being uncertain [8]. Some studies reported that ADC has a poorer prognosis than SCC patients [9,10,11]. For instance, Jung E. et al., in a cohort of 1133 CC patients, reported that the ADC subtype was a significant independent factor for poor overall survival (OS) (*p* = 0.0001). Likewise, Yamauchi M. et al. reported in a smaller cohort that ADC had a significantly poorer prognosis in CC patients (*p* = 0.001) [11]. On the other hand, other studies reported that ADC showed recurrence and survival rates equivalent to those of SCC patients [12,13]. For instance, Winer I. et al. reported, in a small cohort of patients, that the 5-year OS was comparable for ADC and SCC (98.2% and 95.2%, respectively, *p* = 0.369) [14]. Additionally, in a larger cohort of patients, Xie X. et al. reported that there was no difference in the 5-year survival rate between SCC and ADC patients (87.3% vs. 82.4%; *p* > 0.05) [12].

Considering these contradictory data, it is important to find molecular differences that distinguish each subtype based on the gene expression profile and that can be used as prognostic biomarkers for these patients. Some attempts have been made to find molecular differences between ADC and SCC; for instance, Lin W. et al. identified 1733 genes that distinguish ADC from SCC in the lung, esophagus, and cervix; however, they suggested that it is only a catalog of genes and markers that should be studied further [13]. In another study, using microarrays and RT-qPCR assays, Chao A. et al. identified a group of four genes (CEACAM5, TACSTD1, S100P, and MSLN) that were overexpressed in ADC vs. SCC; in this study, CEACAM and TACSTD1 were prognostic factors [15]. Moreover, transcription factors encoding genes PAX6, PDX1, HNF4A, HNF1A, HNF4G, and FOXA3 show higher expression levels in cervical ADC as compared with cervical SCC [16]. These findings demonstrate that it is conceivable to describe different gene profiles for ADC and SCC.

Despite the molecular differences between ADC and SCC mentioned above, the evidence is not conclusive, and this field needs to be explored further. In the present work, our major aim was to identify those molecular profiles that distinguish ADC and SCC and that may have prognosis value. To achieve this, we downloaded transcriptome data corresponding to 309 CC patients from TCGA. We compared ADC vs. SCC and identified 1768 differentially expressed genes (DEGs) between the two histological types. These data were validated in a Mexican-Mestizo independent cohort (GSE56303), and the results were consistent with those obtained with TCGA analysis. Strikingly, 70 genes were concordant—with similar Log2FoldChange values—in both cohorts. Moreover, we utilized three different databases to obtain the possible biological pathways in which these DEGs are involved, and we noticed that SCC tumors have a higher level of activation of critical cancer pathways, such as IL17, JAK-STAT, and Ras, than ADC tumors. Strikingly, of the DEGs, higher GABRB2 and TSPAN8 expression and lower TMEM40 expression were associated with better OS in the Mexican-Mestizo independent cohort (GSE56303). Similarly, when we validated by qPCR the expression of these genes in an independent Mexican cohort, we noted that high expression of GABRB2 and low expression of TMEM40 was associated with better OS.

## 2. Materials and Methods

### 2.1. Analysis of Differential Expression

TCGA data of all CC samples (Stage I-IV) [17,18] were downloaded using the Bioconductor package TCGA biolinks [19]. Differential expression analysis was carried out using the DESeq2 package [20], where an adjusted (adj) *p*-value < 0.01 and a log2FoldChange >2 or <2 were considered significant. Additionally, we analyzed the expression profiles of an independent of 89 Mexico-Mestizo patients diagnosed with local advanced cervical cancer (LACC), obtained from the publicly available database GEO GSE56303 using the R package GEOquery [2,21,22]. This dataset was previously used to obtain an mRNA signature capable of predicting the treatment response in CC patients [2]. The data obtained were normalized using the robust multiarray averaging (RMA) method, and differential expression analysis was carried out using the limma package [23]. An adj *p*-value < 0.05 was considered in order to indicate a significant difference. To construct the heatmaps, a Z-Score transformation was applied to the normalized gene expression values; next, we employed a Hierarchical cluster analysis using the Euclidian distance and the complete-linkage clustering algorithm with the hclust R package.

### 2.2. Cervical Samples

The Mexican independent cohort included 31 CC patients from 2010 to 2013 through the Instituto Nacional de Cancerología of Mexico City (INCan). Of these, fifteen were ADCs and sixteen were SCCs. This study was approved by INCan’s Review Board and Ethics Committee (015/012/IBI-CEI/961/15). All patients of this study agreed and signed the consent form. After a punch biopsy, tumor samples were segmented into two pieces, one for pathological confirmation and another for nucleic acid separation.

### 2.3. RNA Isolation and qPCR

In order to validate differentially expressed genes (DEG), we performed validation by qPCR. Total RNA was extracted from three cuts of FFPE tissue blocks using the RNeasy FFPE Kit (Cat. No. 73504 Qiagen. Hilden, Germany). RNA was quantified in a Qubit 3.0 Fluorometer with a Qubit RNA HS Assay Kit (Cat. No. Q32852. Thermo Fisher Scientific. Waltham, MA, USA). Reverse transcription was performed with 1000 ng of RNA using a High-Capacity cDNA Reverse Transcription Kit (Cat. No. 4374966. Thermo Fisher Scientific. Vilnius, Lituania), following the manufacturer’s recommendations. Quantitative real time PCR was performed with Luminaris HiGreen qPCR Master Mix (Cat. No. K0994) on Step One System (Thermo Fisher Scientific) with primers for GABRB2 Fw: GCACTGGGCAGACTAAGTTGG Rv: GGGTCATTGACACTCTGCGCA, TSPAN8 Fw: TGGTCCTGTATTGCCAGATCG Rv: GGTCTAGCTAGCCGAGACATTT and TMEM40 Fw: CCAGAAGTTTAGGCTGACAGGGT Rv: GGGCTGGTGCCAACATTCAGAC, data were normalized with GAPDH housekeeping gene.

### 2.4. CC Transcriptome and Pathway Analyses

Pathway analysis of the differentially expressed genes of both cohorts (TCGA and Mexican-Mestizo cohort) was assessed using a Gene Set Enrichment Analysis (GSEA) with the Webgestalt platform [24], where a *p*-value < 0.05 was considered to indicate a significant difference.

### 2.5. OS Analysis

We obtained the clinical data from the 309 cervical tumors from the TCGA, the 89 patients from the Mexican-Mestizo cohort and the 31 patients in the Mexican independent cohort. For each cohort, patients were divided in two groups: high and low, depending in the median expression of each gene. Next, the OS of each group was evaluated using the Kaplan-Meier method, and the statistical significance of survival differences was determined with the log-rank test. Univariate and multivariate Cox proportional hazard regressions were assessed using the survival package, implemented in the R language. A *p*-value < 0.05 was considered as significant. OS was defined as the time diagnosis to the date of death or last contact. It is important to explain that for the OS analyses, we possessed complete 5-year follow-up information for the Mexican-Mestizo cohort and the Mexican independent cohort. However, for the TCGA patients, we used only the data that were available, since follow-up information was not available for all patients

## 3. Results

### 3.1. Determination of the Molecular Profile Distinguishing ADC and SCC among CC Patients from the TCGA Database

Our first aim was to determine molecular differences between SCC and ADC. Thus, we analyzed the transcriptomes of the TCGA CC cohort. This dataset contains information on 260 SCC and 49 ADC tumors from patients of different ethnicities. The clinical characteristics are shown in Table 1. It should be noted that the clinical data of the TCGA cohort only contained the HPV information from 22 patients (7%) (Table S1), whereas 63 patients (70.78%) from the Mexican-Mestizo cohort were positive to HPV infection, along with all patients from Mexican independent cohort (Table S2).

**Figure 1:**
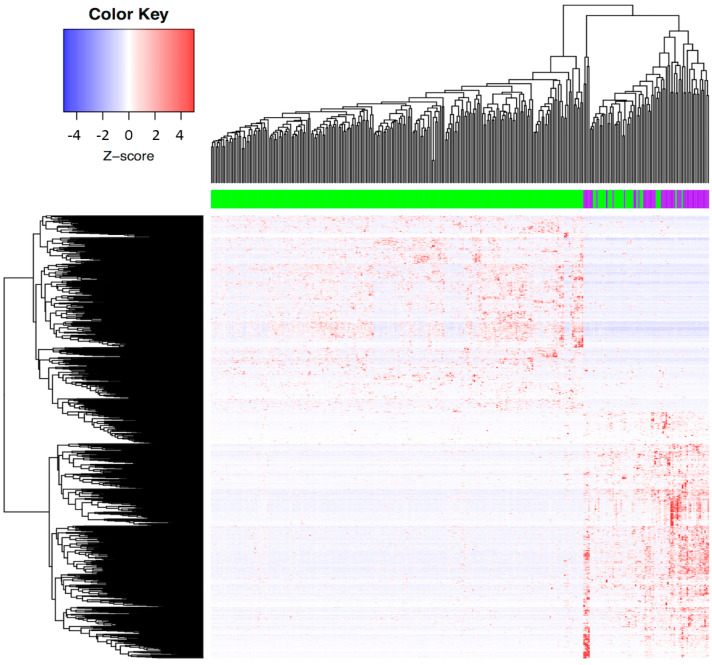
**Molecular profile distinguishing cervical ADC and SCC in CC patients from the TCGA Database.** Each column represents a patient with CC, and each row represents the expression of a gene. Gene expression changes are represented in blue (upregulated), red (downregulated), and white (no significant change or the absence of data). Patients with SCC are represented in green and ADCs in purple. There were 1768 unique DEGs between SCC and ADC tumors (adj *p*-value < 0.05, log2FoldChange >2 or <2), and most of the ADC tumors were clustered together.

The results showed 1768 unique DEGs between SCC and ADC (adj *p*-value < 0.05, log2FoldChange >2 or <2) (Table S3), where most of the ADC tumors were clustered together (Figure 1). This finding confirms the existence of molecular differences between the two histological types of cervical tumors. A list of the top DEGs between the two histological types is depicted in Table 2.

### 3.2. Validation of the Molecular Profile Obtained from the TCGA Database in a Mexican-Mestizo Independent Cohort

**Figure 2:**
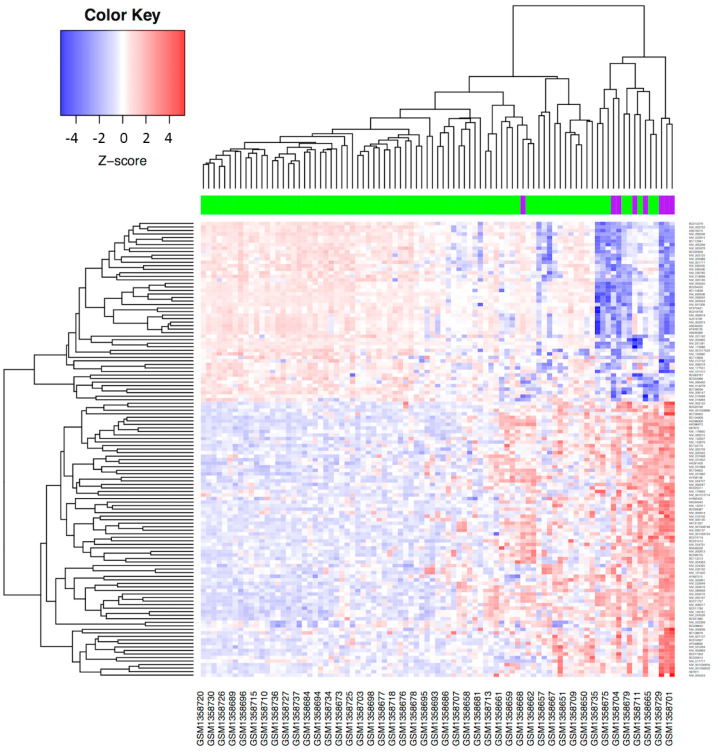
**Molecular profile distinguishing ADC and SCC in CC patients from the Mexican-Mestizo cohort.** Each column represents a patient with CC, and each row represents the expression of a gene. Gene expression changes are represented in blue (upregulated), red (downregulated) and white (no significant change or the absence of data). Patients with SCC are represented in green and ADCs in purple. In total, 130 transcripts separated Mexican-Mestizo CC patients into ADC vs. SCC subgroups (adj *p*-value < 0.05).

To validate the differential expression profile between SCC and ADC obtained from the TCGA data analysis, we utilized an online, publicly available transcriptome dataset (GSE56303) [2]. This dataset only includes a Mexican-Mestizo population, which comprises 89 CC patients, 81 of whom had SCC and 8 of whom had ADC. The analysis of DEGs between the two histological types revealed a profile of 130 transcripts that separated CC patients into ADC vs. SCC subgroups (*p* adj < 0.05) (Table S4). Strikingly, the 130 differentially expressed transcripts corresponded to 88 unique genes, of which 52 were overexpressed and 36 were downregulated (Figure 2). HNF1A-AS1, SPINK1, EPS8L3, PROM1, and TM4SF5 were the most overexpressed genes, whereas CLCA2, CALML3, PKP1, GPR87, and SPRR2A were the most downregulated genes in ADC (Table 3). This finding suggested a histology-driven molecular profile.

**Figure 3:**
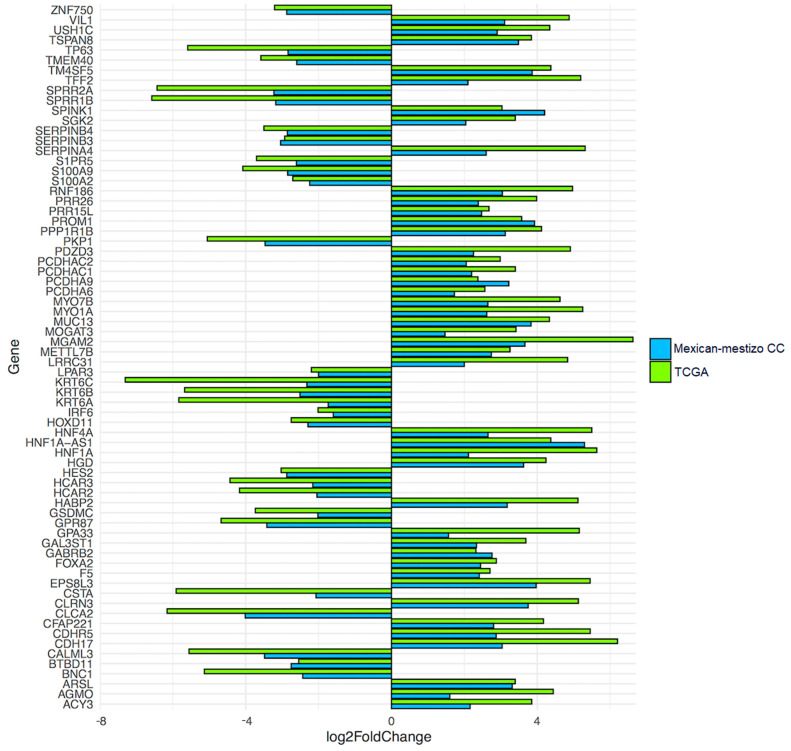
**DEGs in the Mexican-Mestizo CC dataset and the TCGA dataset are concordant.** Blue represents the 88 unique DEGs in the Mexican-Mestizo CC dataset. Green represents the 70 DEGs from the TCGA dataset.

Notably, of the 88 unique genes differentially expressed between ADC and SCC patients in the Mexican-Mestizo cohort, 70 were also differentially expressed in the TCGA cohort and even presented a similar log2FoldChange values (Figure 3) (Table S5). These findings show a high concordance of the molecular differences of the two histological types between cohorts.

### 3.3. Signaling Pathway Enrichment Analysis in DEGs between ADC and SCC Subtypes

**Figure 4:**
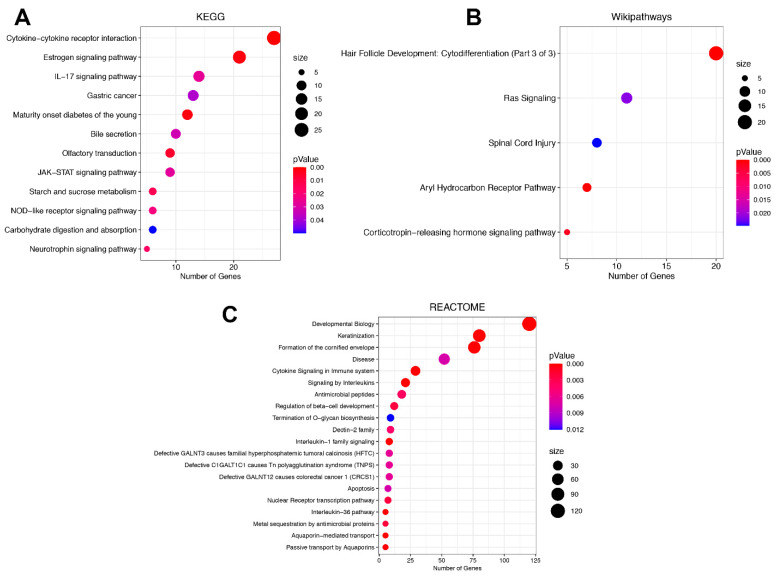
**Signaling pathway enrichment analysis of DEGs between ADC and SCC patients performed with the WebGestalt platform.** (**A**) Enriched pathways in the KEGG database analysis. (**B**) Enriched pathways in the WikiPathways database analysis. (**C**) Enriched pathways in the Reactome database analysis. The size of the dots represents the number of DEGs in the pathway, while the dot color represents the significance of the analysis, where red shows a more significantly enriched pathway.

Once we recognized that there was a differential expression profile between SCC and ADC, we decided to determine which signaling pathways the DEGs were involved in. For this purpose, we performed an analysis using the Webgestalt platform exploiting three different databases—the Kyoto Encyclopedia of Genes and Genomes (KEGG), WikiPathways, and Reactome databases—to obtain a global pathway assessment of the deregulation of both subtypes. It is important to mention that each database has a different set of manually curated genes; thus, the results could differ between them. In the KEGG database analysis, the pathways with the highest number of deregulated genes were the cytokine-cytokine receptor interaction, estrogen signaling and IL-17 signaling pathways. Interestingly, we also found that the JAK-STAT signaling pathway was significantly enriched in this analysis (Figure 4A). When assessing the WikiPathways database, we found that Ras signaling was highly enriched in DEGs (Figure 4B). In the Reactome database analysis, we also found deregulation in pathways associated with the immune response, such as cytokine-cytokine receptor interaction, and again, the estrogen signaling pathways, and the IL-17 and JAK-STAT signaling pathways (Figure 4C). Interestingly, our results show that SCC tumors have a higher level of activation of critical cancer pathways, such as IL17, JAK-STAT, and Ras, than ADC tumors (Figure 4A–C). Overall, we assessed different databases and observed enrichment of multiple signaling pathways that are commonly dysregulated in cancer, suggesting that our results are highly reliable and independent of the database used.

**Figure 5:**
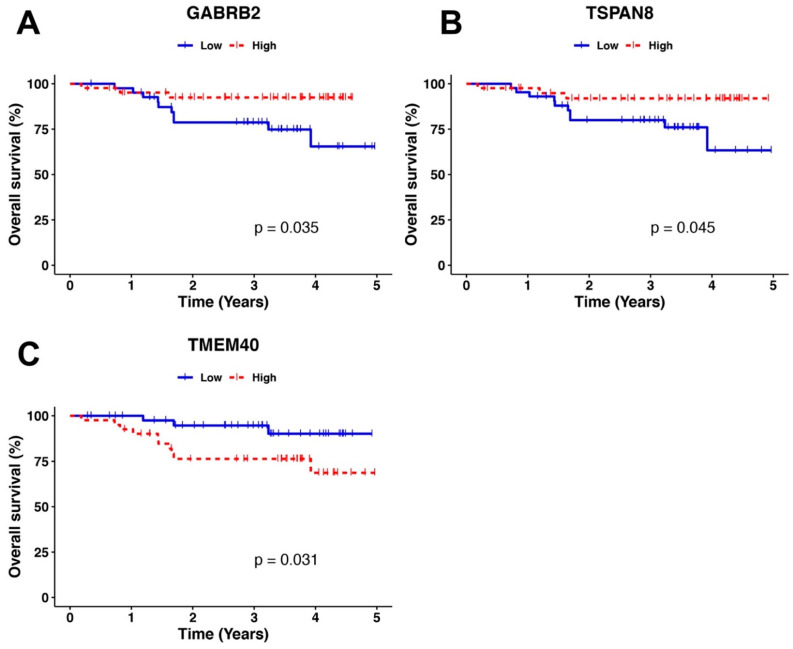
Kaplan-Meier OS analysis of Mexican-Mestizo patients. (**A**) Differences in the OS of patients with high and low expression of GABRB2. (**B**) Differences in the OS of patients according to TSPAN8 expression. (**C**) Differences in the OS of patients according to TMEM40 expression. Blue lines represent patients with low gene expression, and red lines represent patients with high gene expression.

**Figure 6:**
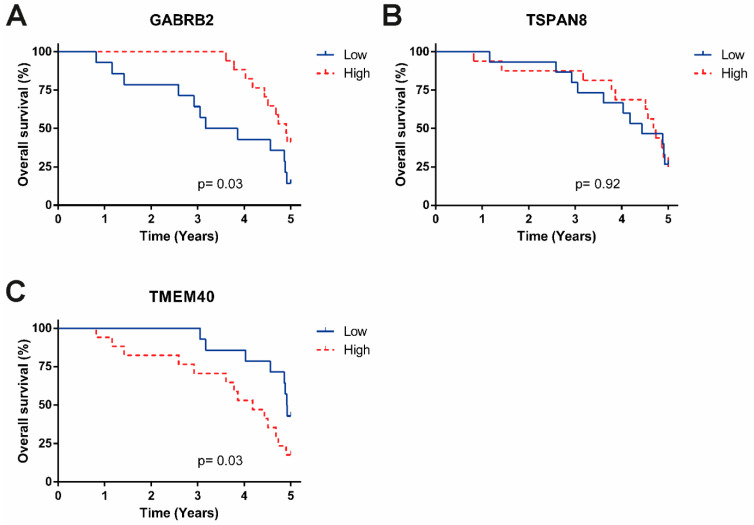
Kaplan-Meier OS analysis of independent Mexican cohort. (**A**) Differences in the OS of patients with high and low expression of GABRB2. (**B**) Differences in the OS of patients according to TSPAN8 expression. (**C**) Differences in the OS of patients according to TMEM40 expression. Blue lines represent patients with low gene expression, and red lines represent patients with high gene expression.

To determine whether the DEGs between SCC and ADC are correlated with OS, we used survival data from the TCGA, Mexican-Mestizo, and independent Mexican cohorts. The Kaplan-Meier curves showed no significant difference between the survival of SCC vs. ADC patients in the TCGA dataset (Figure S1). However, when we used the survival data from the Mexican-Mestizo cohort, the Kaplan-Meier analysis showed that high expression of the DEGs GABRB2 and TSPAN8 and underexpression of TMEM40 were associated with favorable OS (Figure 5). Moreover, the univariate and multivariate cox-regressions, using these genes, HPV infection, and tumor histology as covariates, showed that only TMEM40 and GABRB2 were able to independently differentiate the OS of the patients, whereas HPV infection or tumor histology were not significant factors for the OS (Likelihood ratio test *p* = 0.05, Wald test *p* = 0.09, Logrank test *p* = 0.06) (Table 4). Additionally, we performed qPCR to validate the expression of the DEGs GABRB2, TSPAN8, and TMEM40 in the 31 patients of the independent Mexican cohort. Using the median expression value of each gene validated, patients were sorted into two groups, those with high and low expression. Similarly to Mexican-Mestizo cohort, Kaplan-Meier curves showed that Mexican patients with high expression of GABRB2 and underexpression of TMEM40 were associated with favorable OS (Figure 6).

## 4. Discussion

It is well described that persistent infection with HR-HPV is the causal factor in the development of CC [25]. Several reports have described that HPV16 is more frequent in SCCs, and HPV18 and HPV45 in ADCs [5,6,7]. Hence, it is to be expected that there are molecular and clinical differences in both histologic subtypes. In this regard, several reports have described controversial data about the prognosis of CC patients with ADC and SCC. For instance, some of them have reported that ADC is associated with a poorer prognosis than SCC [9,10,11]. However, other studies described that ADC and SCC displayed equivalent survival outcomes [11,12]. For instance, in a small cohort of patients, the 5-year OS was comparable for ADC and SCC (98.2% and 95.2%, respectively, *p* = 0.369) [14]. Additionally, in a larger cohort of patients, it was reported that there was no difference in the 5-year survival rate between SCC and ADC patients (87.3% vs. 82.4%; *p* > 0.05) [12]. In this respect, researchers have focused on identifying molecular differences unique to one or another histological subtype so that they can be used as prognostic biomarkers for these patients. Nonetheless, this field needs to be investigated further. In this work, we identified a molecular profile of DEGs between ADC and SCC, in CC patients from TCGA and in the independent Mexican-Mestizo cohort. Despite the fact that the TCGA database includes patients from different ethnic groups and all clinical stages, we detected that, of the DEGs, 70 overlapped with genes from the Mexican-Mestizo database and even displayed a similar log2FoldChange values, thus demonstrating a high concordance in the molecular differences between the two histological types in the datasets. Similarly, other studies have demonstrated molecular differences between SCC and ADC histologic types of CC patients. For instance, Lin W.E. et al. analyzed TCGA data and reported 1733 DEGs between ADC and SCC from CC patients [13]. Likewise, Chao A. et al., through microarray assays, revealed a profile of DEGs between ADC and SCC samples from CC patients; TSPAN-3, CEACAM5, TACSTD1, MSLN, and S100P were of particular interest, as they were overexpressed in ADC [15]. Moreover, transcription factors encoding genes PAX6, PDX1, HNF4A, HNF1A, HNF4G, and FOXA3 show higher expression levels in cervical ADC as compared with cervical SCC [16]. These findings confirm that there are molecular differences between ADC and SCC.

The molecular differences between ADC and SCC also have an impact on signaling pathways that promote disease. In this work, the KEGG, WikiPathways, and Reactome databases were employed to elucidate these differences. We found that SCC tumors have a higher level of activation of critical cancer pathways, such as IL-17, JAK/STAT, and Ras signaling, compared to ADC tumors. In this respect, it is well-known that IL-17 is the central cytokine of the Th17 response during persistent infection with HR-HPV; this response triggers chronic inflammation of a long duration and the production of IL-17, among other pro-inflammatory cytokines, hence creating a favorable environment for the tumor development associated with associated with poor CC prognosis [26,27,28]. Additionally, Punt S. et al. reported that the predominant cell type expressing IL-17 in SCC CC is the neutrophilic granulocyte and directly contributes to tumorigenesis [29,30]. Although there are no studies that evaluate the correlation between IL-17 and its histological subtypes (ADC and SCC), we observed that SCC has a higher level of IL-17 pathway activation. JAK/STAT signaling was another deregulated pathway in our study. This pathway has been found involved in proliferation, invasion, survival, inflammation, and immunity in CC patients [31]. Strikingly, there is a connection between JAK/STAT pathway and Th17 cells, since JAK/STAT pathway is necessary for the differentiation of Th cells [32]. The aberrant signaling of JAK/STAT pathway has not been associated with any histological subtype (ADC vs. SCC). However, it is expected that this pathway is altered in both subtypes, nonetheless we were able to distinguish that it is enriched in the SCC subtype, which is an interesting fact that needs to be analyzed in depth. Another pathway enriched in our study was Ras signaling. Aberrant activation of this pathway is common in several cancers, including CC, which often results from the presence of mutations and amplifications of KRAS. For instance, Zou Y. et al. reported that the frequency of the KRAS mutation ranged from 8.0–17.5% in cervical ADC [33], to absent or rare in SCC, which suggested that KRAS mutations are frequent and might be a driving factor for the development of cervical ADC but not SCC [34,35]. However, in our study, we observed that the Ras pathway has a higher level of activation in SCC than ADC, which may be due to mutations or alterations in other elements of the pathway [33]. According to the fact that these pathways are more overexpressed in SCC than in ADC, it leads us to speculate that SCC seems to be more aggressive than ADC; however, the candidate pathways and genes for distinguishing the subtypes need to be further verified.

A main focus of this work was to identify potential genes associated with the prognosis of ADC or SCC, and we generated Kaplan-Meier survival curves with the Mexican-Mestizo, Mexican, and TCGA patient’s clinical data. We noticed that GABRB2 and TSPAN8 overexpression and TMEM40 under-expression were associated with favorable OS, in the Mexican-Mestizo and independent Mexican cohorts. Regarding the role of these genes in cancer, there has been only one report associating GABRB2 overexpression with lymph node metastasis in thyroid cancer [36]. TSPAN8 expression is correlated with a poor prognosis in breast cancer [37], renal cell carcinoma [38], and pancreatic cancer [39]. However, its role in CC had not yet been studied. On the other hand, TMEM40 has been poorly studied in cancer, but a study proposed that this protein plays a crucial role in proliferation and apoptosis via the p53 signaling pathway [40] and may be a potential diagnostic biomarker for bladder cancer [41]. In our study, we noticed that the expression levels of GABRB2, TSPAN8, and TMEM40 were not associated with the OS of TCGA CC patients (Figure S1). In this respect, it is well-known that the TCGA database contains incomplete or no information regarding the survival of many patients. This often occurs because almost all of the patients are untreated and thus have no response data and short follow-up periods [17]. Moreover, Lin W.E. noted that the TCGA database is interesting because it includes patients of different ethnicities; however, although TCGA datasets are generally large, they may not be representative of the general population [13]. It is possible that for this reason, in our analysis, we did not find any association between the expression of these genes and OS with the TCGA CC patients. Likewise, it is reasonable to assume that there is a consistent molecular profile for each histological subtype. Taken together, it is important to note that we found molecular differences between the two histologies (ADC vs. SCC); nevertheless, in order to identify a specific prognostic biomarker for each subtype it is necessary to expand the sample of ADCs, since, in the cohorts used, including the TCGA cohort, the number of ADCs is limited. Therefore, the correlation of these genes with the survival or prognosis of patients’ needs to be studied meticulously.

## 5. Conclusions

These findings are relevant since they show a high concordance of molecular differences for ADC versus SCC between independent cohorts, independently of HPV type, thus opening a window of opportunity to identify new prognostic biomarkers by histological type. Nonetheless, further studies are required to define these findings.

## Figures and Tables

**Table 1:** Clinical characteristics of the TCGA cohort.

Characteristics		Total (*n* = 309)	SCC (*n* = 260)	ADC (*n* = 49)
Ethnicity	Hispanic or Latino	24 (7.76%)	21 (8.07%)	3 (6.12%)
	Not Hispanic or Latino	171 (55.33%)	145 (55.76%)	26 (53.06%)
	Not reported	114 (36.91%)	94 (36.15%)	20 (40.81%)
FIGO Stage				
	Stage I	163 (52.75%)	129 (49.61%)	34 (69.38%)
	Stage II	70 (22.65%)	63 (24.3%)	7 (14.28%)
	Stage III	46 (14.88%)	43 (16.53%)	3 (6.12%)
	Stage IV	21 (6.79%)	17 (6.53%)	4 (8.16%)
	Not reported	9 (2.91%)	8 (3.07%)	1 (2.04%)

SCC, squamous cervical carcinoma; ADC, adenocarcimoma of cervix.

**Table 2:** Top 15 DEGs in ADC versus SCC in the TCGA dataset.

Over-Expressed		Under-Expressed	
Gene	log2FC	Gene	log2FC
REG4	8.41	CRNN	−9.38
TM4SF4	8.27	KRT14	−8.57
TAAR1	7.54	TMPRSS11B	−8.17
FABP1	7.52	CLEC2A	−8.07
SLC17A4	7.42	SPRR2E	−7.84
ANKRD40CL	7.32	TMPRSS11BNL	−7.82
REG1A	6.77	LCE3E	−7.78
REG1B	6.65	SERPINB12	−7.78
MGAM2	6.63	KPRP	−7.73
LOC105375166	6.57	LCE3D	−7.66
MUC17	6.57	TMPRSS11A	−7.56
CCL15	6.33	SPRR2G	−7.33
SLC39A5	6.26	KRT6C	−7.31
LOC107105282	6.23	LINC02582	−7.30
CDH17	6.20	KRT1	−7.26

**Table 3:** Top 15 DEGs in ADC versus SCC in the Mexican-Mestizo CC dataset.

Overexpressed		Underexpressed	
Gene	log2FC	Gene	log2FC
HNF1A-AS1	5.30	CLCA2	−4.02
SPINK1	4.20	CALML3	−3.48
EPS8L3	3.97	PKP1	−3.47
PROM1	3.93	GPR87	−3.42
TM4SF5	3.86	TP63	−3.41
EPS8L3	3.86	SPRR2A	−3.23
MUC13	3.83	SPRR1B	−3.18
USH1C	3.77	SERPINB3	−3.17
CLRN3	3.75	GPR87	−2.98
HGD	3.73	ZNF750	−2.88
MGAM2	3.66	SERPINB4	−2.86
HGD	3.63	S100A9	−2.85
TSPAN8	3.48	TP63	−2.84
ARSL	3.31	BTBD11	−2.75
HNF4A	3.25	KRT6C	−2.73

**Table 4:** Univariate and multivariate cox regressions in the Mexican-Mestizo CC dataset.

		**Univariate Analysis**		**Multivariate Analysis**	
	**Overall Survival**	**HR (95% CI)**	***p*** **-Value**	**HR (95% CI)**	***p*** **-Value**
TMEM40	High vs. low expression	0.27 (0.073–0.97)	**0.045**	0.32 (0.08–1.22)	0.096
GABRB2		3.7 (1–13)	**0.049**	2.93 (0.78–10.9)	0.108
TSPAN8		3.5 (0.95–13)	0.059		
HPV	Negative vs. positive	1.1 (0.31–4.1)	0.86	1.05 (0.28–3.84)	0.937
Hystology	Adenocarcinoma vs. epidermoid	1.2 (0.15–9)	0.88		

## Data Availability

All data used in the present study are publicly available.

## Supplementary Materials

The following supporting information can be downloaded at: https://www.mdpi.com/article/10.3390/curroncol29070372/s1, Table S1. Clinical data of the TCGA cohort, Table S2. HPV data from Mexican-Mestizo cohort, Table S3. Differential expressed genes between the two histological types in TCGA cohort, Table S4. Differential expressed genes between the two histological types in Mexican-mestizo cohort (GSE56303), Table S5. 70 DEGs concordant between TCGA and Mexican-mestizo cohort, Figure S1. GABRB2, TSPAN8 and TMEM40 Overall survival of TCGA CC patients.
