# Supplementary figures and images for: Molecular Differences between Squamous Cell Carcinoma and Adenocarcinoma Cervical Cancer Subtypes: Potential Prognostic Biomarkers
Source: Curr Oncol. 2022 Jul 5;29(7):4689–702. doi: 10.3390/curroncol29070372 (PMC9322365; doi:10.3390/curroncol29070372)

Figure S1. GABRB2, TSPAN8 and TMEM40 Overall survival of TCGA CC patients.

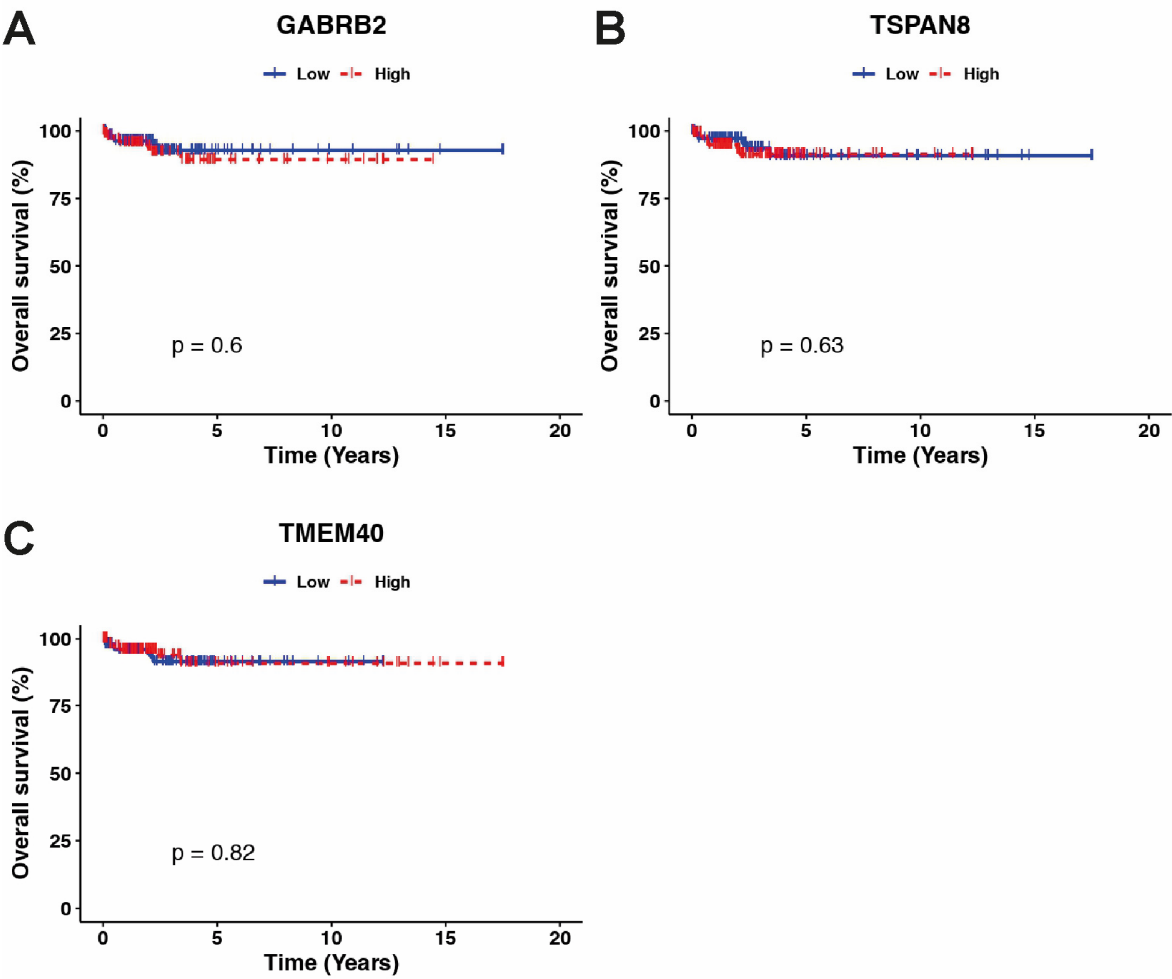

Supplement: Supplementary file 1 [file curroncol-29-00372-s001.zip › Figure S1.pdf]
